# Information and communication technologies-based interventions for children with autism spectrum conditions: a systematic review of randomized control trials from a positive technology perspective

**DOI:** 10.3389/fpsyt.2023.1212522

**Published:** 2023-07-20

**Authors:** Ileana Scarcella, Flavia Marino, Chiara Failla, Germana Doria, Paola Chilà, Roberta Minutoli, Noemi Vetrano, David Vagni, Loris Pignolo, Marcella Di Cara, Carmela Settimo, Angelo Quartarone, Antonio Cerasa, Giovanni Pioggia

**Affiliations:** ^1^Institute for Biomedical Research and Innovation (IRIB), National Research Council of Italy (CNR), Messina, Italy; ^2^Faculty of Psychology, International Telematic University Uninettuno, Rome, Italy; ^3^Kore University of Enna, Enna, Italy; ^4^Department of Cognitive, Psychological Science and Cultural Studies, University of Messina, Messina, Italy; ^5^S'Anna Institute, Crotone, Italy; ^6^IRCCS Centro Neurolesi Bonino-Pulejo, Messina, Italy; ^7^Pharmacotechnology Documentation and Transfer Unit, Preclinical and Translational Pharmacology, Department of Pharmacy, Health and Nutritional Sciences, University of Calabria, Rende, Italy

**Keywords:** ICT—information and communication technologies, social abilities, autism, computer, tablet, social robots

## Abstract

Information and communication technologies (ICTs) have become more widely used in the past years to help people with autism spectrum conditions (ASC). Serious games embedded into computers or tablets, as well as social robots, are the most employed ICT-related tools that are appealing to and appropriate for autistic children. The goal of ICT applications is to enhance behavioral abnormalities associated with ASC while also creating an interactive link between one person and one computer. Comparatively, to human-based therapy, ICT tools aid to inspire autistic children by providing predictability and regularity of tasks. Regaining social skills is the primary behavioral goal for which ICT tools have been designed and implemented. In the past several years, many studies have been created to show how effective it is at improving targeted behaviors. However, only a small number of researchers have used an RCT approach to evaluate its effectiveness. In this systematic review, we only included RCT studies where ICT technologies were used to help children with ASC in improving their social skills. Only 14 RCT studies satisfied the criteria and 12 described significant improvements, showing how the use of technology in educational contexts produced better improvement in developing several social skill facets with respect to the traditional face-to-face approach. Some studies used interventions and outcome measures focused on the core ASC symptoms, but many others addressed neurocognitive functions directly, like social cognition or emotional regulation, while other more general functions such as language or adaptive behaviors. We propose a classification based on processes and outcome measures to foster future research in this specific area of research. The behavioral intervention mediated by technological tools such as computer-based, tablet, and social robotics, undoubtedly provides a comfortable environment that promotes constant learning for people with ASC. Evidence provided in this review highlights the translational potential of this field of study in primary care practice and educational settings.

## 1. Introduction

The knowledge society relies on information and communication technologies (ICT) as they enable people to acquire new languages and mindsets as well as improve their social and academic status (1). According to Riva et al. 2012, ICT can be used as a technology to modify and enhance our personal experience with the aim of increasing wellness, and creating strengths and resilience in individuals, organizations, and society. This “ICT approach” is based on Positive Technology, a specific subfield of Positive Psychology, which uses technology to foster well-being and the quality of one's own experience (2). Positive psychology focuses on the factors that support positive functioning and adaptive behaviors, allowing people and societies to thrive. ICT is especially useful in clinical psychology and the (re)habilitation of people with physical, mental, and neurological disabilities in this context. Indeed, ICT provides tools, network services, hardware, and software to help persons with disabilities to overcome the challenges they face, promote independence, and achieve their full potential. As a result of this evidence, the research and design of technologies to be applied in the field of clinical psychology have grown considerably and today represent a valid tool for the promotion of health and well-being (3–5).

In the field of clinical psychology, the literature describes several ICT-based interventions, mainly focused on some neurological conditions, such as stroke (6) and dementia (7), where tablets, computers, or robots are used to assess, maintain, or improve cognitive functions, or to mitigate behavioral disorders (8). Applications for helping children with a diagnosis of autism spectrum disorder (ASD) are another area where ICT is expanding at a rapid rate. In order to make it easier for the reader to locate important information in this field of study, we grouped the ICT tools in this review into three main areas: (a) Computer-based serious games; (b) Tablet-based serious games; and (c) Robotic devices (9).

In this paper, we adopt a biopsychosocial approach to understand the experiences and needs of autistic persons. We acknowledge that autism is a complex and heterogeneous phenomenon that involves biological, psychological, and social factors. We also recognize that autism is a part of the identity and culture of many autistic persons, who may prefer to be referred to as such rather than as persons with autism (10, 11). Accordingly, we will use the term “Autism Spectrum Disorder” (ASD) only when we refer to the medical diagnosis of autism, based on the DSM-5 or the ICD-11 criteria, while we will use “Autism Spectrum Conditions” (ASC) when we describe the condition itself. In other cases, we will use the term “autistic person” or “autistic persons” to refer to the social issues and experiences of autistic persons, honoring their self-identification and agency. We think that this language choice shows our respect and appreciation for the diversity and dignity of autistic persons. Our research group is composed of people with different backgrounds in psychology, medicine, engineering, mathematics, and one of us identifies as an autistic person. His positionality and perspective have contributed to our research process and analysis. Our main focus is the application of technology for people on the autism spectrum.

Autism spectrum condition (ASC) is a term that covers a variety of ways that people experience and interact with the world differently. People with ASC have diverse strengths and challenges in social communication and may have specific interests or preferences. Some children with ASC may find it hard to connect with others or to express themselves in ways that are expected or accepted by society. They may talk about topics that are not related to the conversation, or only share their own passions. These children may need more support and understanding to develop their social skills and confidence. If they don't get the support they need, they may feel anxious, depressed, or lonely (12). There are many ways to help children with ASC learn and practice social skills. One of them is applied behavior analysis (ABA), which is a way of teaching new behaviors based on positive reinforcement. ABA can be used to teach children how to play with others, such as pretending or acting out stories (13) or (14, 15). Another way is to involve other children who are not autistic in the learning process. These children can be friends, classmates, or siblings who can help the autistic child feel more comfortable and engaged in social situations. Some of the methods that use this approach are peer proximity, which means putting the autistic child near another child who is not autistic (16); peer training, which means teaching the other child how to interact with the autistic child in a supportive way; and peer initiation, which means encouraging the other child to start and continue a conversation with the autistic child. Peer modeling, which means showing the autistic child how to behave socially by example, is not enough by itself to help the autistic child learn social skills (17). Some newer methods that also show promise are ESDM (18) and social-ABC (19). This last one is a method that involves the parents or caregivers of the autistic child in teaching them social skills using ABA principles and adapting them to the child's developmental needs.

However, many autistic people and their allies oppose many behavioral interventions and specifically ABA for various reasons. One of the main criticisms of ABA is that it is based on a deficit model of autism, which views autistic traits as problems to be fixed or eliminated. This can lead to a loss of identity, self-esteem, and autonomy for autistic people. Another criticism of ABA is that it can cause trauma to autistic people, when it involves aversive stimuli and even when ABA uses only positive reinforcement, it can still be perceived as coercive and stressful (20–22). Recently the ABA community is working to fix many of those issues and endorsing more respectful and ethical positions. Nevertheless, many people in the autistic community think that behavioral interventions usually do not measure meaningful outcomes for autistic people, such as quality of life, happiness, or well-being, but only focus on observable behaviors that are deemed socially acceptable. We believe that it is important to distinguish between the utility and science of behavioral modification and its goals and application. We would like to show how a positive technology approach can help with that.

ICT interventions based on positive technology are different from behavioral intervention because they do not aim to normalize autistic people, but to remove barriers that prevent them from accessing education, communication, and socialization. ICT interventions use devices such as computers, robots, virtual reality, or tactile and auditory prompts to enhance the learning and well-being of autistic people, according to their strengths, interests, and preferences (23). Indeed, these tools give physicians a variety of working support and enable the production of real-world scenarios in a controlled environment (24). Social skills programs should be tailored to address these challenges. Moreover, autistic children may find ICT applications especially appealing and engaging, according to some studies (25). Scientific evidence suggests that programs that provide direct and immediate feedback, personalized reinforcement, and teacher support can enhance the effectiveness of technology (26, 27).

In addition, touch screens and tangible hardware can facilitate the use of the web, online games, and virtual worlds to improve social learning skills (28). Virtual reality applications, for example, offer autistic individuals the opportunity to improve their cognitive and social skills through safe and realistic situations, while computer, tablet, or mobile applications can be promising training tools as long as accompanied by human assistance (29, 30). Current research has also shown that virtual reality interventions lead to faster and more stable acquisition of social skills over time (31). Finally, social robots with vocal technology can play a significant role in helping autistic children develop social skills (29). However, the aforementioned technologies must be thoroughly examined regarding the skills transferred to the real-life experiences of autistic individuals. Furthermore, taking into account individual differences should be a priority in the design of innovative technologies, such as through the implementation of adaptive systems or through the combination of various strategies (32).

In the last few years, new studies have been made for demonstrating the impact of ICT tools in improving social skills in autistic children, but until then the vast majority has received limited empirical clinical validation. For this reason, we sought to perform, for the first time, a synthesis of recent advances in this challenging field of study, where an RCT design approach has been employed to evaluate the effectiveness of ICT-mediated applications with respect to traditional behavioral approaches. The final aim is to demonstrate the translational potential of this field of study in primary care practice and educational settings.

## 2. Methods

**Figure 1:**
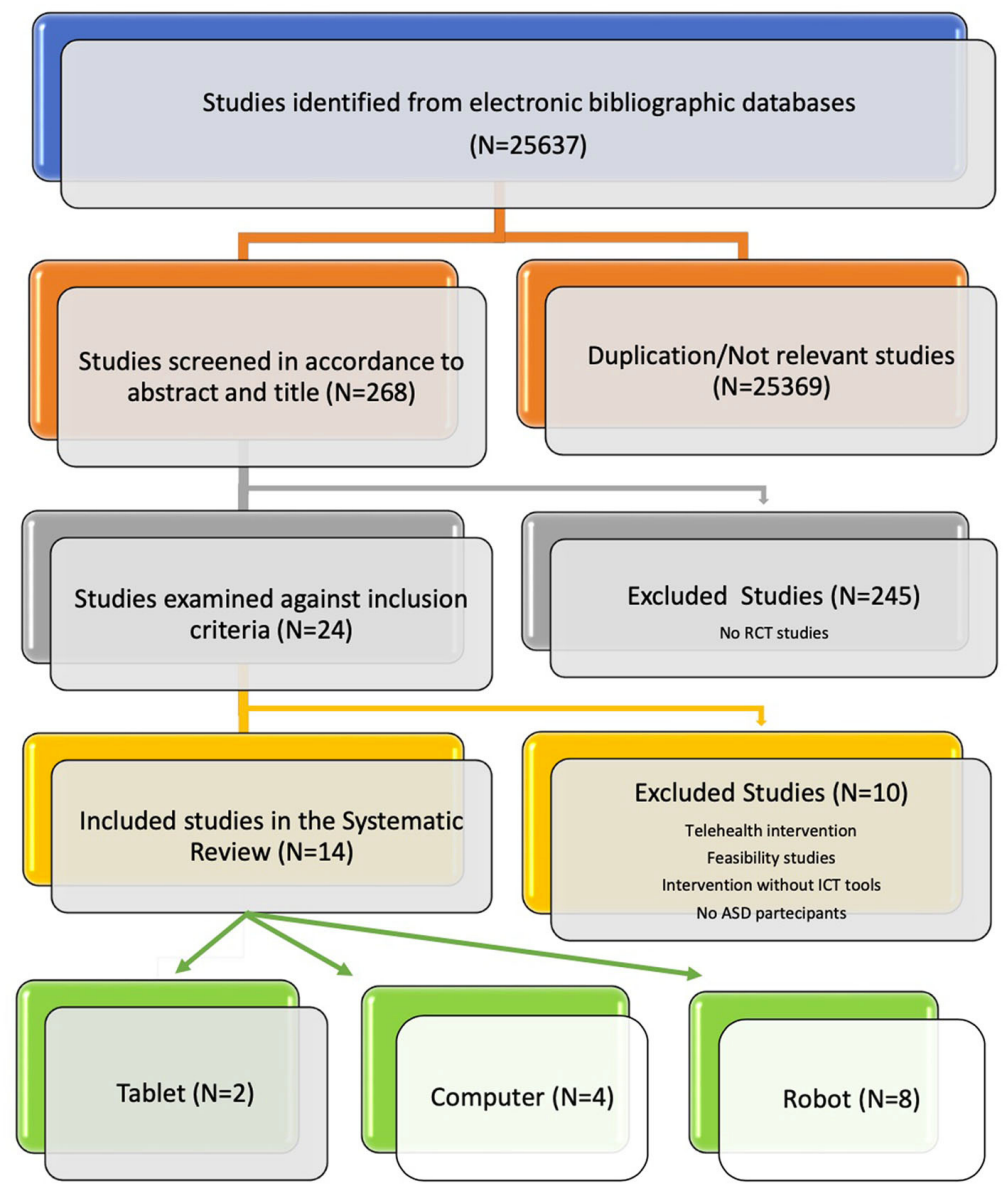
The PRISMA analysis.

This review was planned and conducted in accordance with PRISMA (Preferred Reporting Items for Systematic Reviews and Meta-Analyses) guidelines (33). Articles published between 2011 and 2022 were reported using electronic bibliographic databases such as PubMed, Science Direct, Google Scholar. To improve the search strategy, keywords including “text words” and MeSH were used. The search terms incorporated the following keywords: “Information and Communication Technologies, Autism, Autism Spectrum Disorder, social skills, social-communication skills, socio-emotional competencies, Tablet, Computer, Robot” in titles, abstracts and full text (Figure 1). After the initial web search, duplicate items and non-relevant studies among databases were removed. To reduce the risk of bias, two authors (I.S. and A.C.) independently screened paper abstracts and titles and analyzed the full papers that met the inclusion criteria, as suggested by the PRISMA guidelines. The reference lists of examined full-text papers were also scrutinized for additional relevant publications. The analysis of literature was conducted by one clinical psychologist (I.S.), one behavioral therapist (R.M.) and one statistical scientist (D.V.).

Criteria for including or excluding papers were determined a priori. Papers were considered for inclusion only if they: (a) were written in full-text English language in a peer review journal; (b) were published from 2011 to the end of search December 31, 2022, (c) included autistic children; (d) used randomized controlled trials (RCTs); and (e) used ICT-based interventions. Articles were excluded if they were: (a) telehealth interventions and feasibility studies; (b) unpublished dissertations, book chapters, and conference papers. We further removed all articles not directly interested in evaluating the effects of ICT tools in improving social skills (i.e., emotional and social-communication skills, joint attention, play behaviors, gesture use) in children with an ASD diagnosis. The data collected from each article were categorized as information on the first author and year of publication, the ICT system, the size of cohorts, the modalities of intervention, the experimental procedures, the outcomes measures, and the main results.

A more thorough search of the literature for papers before 2011 revealed no studies that met our inclusion criteria.

## 3. Results

### 3.1. Study selection

The electronic bibliographic databases search strategy of five databases retrieved 25,637 studies. After screening titles/abstracts and adjusting for relevance *n* = 269 studies remained after the reviewing process. From this group, 245 studies were further excluded because they were not randomized controlled trials. In the second phase, 10 studies were excluded because they did not fulfill inclusion criteria. Indeed 4 studies were removed because the intervention was not specifically addressed to children with autism spectrum disorder, 3 studies did not use ICT tools, 2 were feasibility studies and one was a telehealth intervention. Finally, 14 articles were included in this review (Figure 1).

### 3.2. Neuropsychological assessment of social skills

Considering the overall papers selected for this systematic review, a wide range of neuropsychological tests have been used to assess social skills in autistic children. In Table 1, we report every single test employed together with the respective skill investigated.

**Table 1:** Summary of the neuropsychological tests employed to assess social skills in asd children in ict-related clinical trials.

**Tests**	**Facet n°1**	**Facet n°2**	**Facet n°3**	**Facet n°4**	**Facet n°5**	**Facet n°6**	**Facet n°7**	**Facet n°8**	**Facet n°9**	**Facet n°10**
ADOS	Autism Diagnostic									
BASC-2-PRS	Generic									
CAM-C	Affective expression recognition	Affective Prosody Recognition								
CARS	Autism Diagnostic									
CGI-I	Generic									
CSBS	Communication	Gesture	Facial Expression	Joint Attention	Play Behavior	Symbolic Behavior	Play			
ECBI-P:	Generic									
EDRS	Affective expression recognition									
ELT	Comprehension of emotional lexicon	Contextual affect understanding								
ERC	Emotion Regulation									
ERSSQ-P	Emotion Regulation	Social Skills								
ERSSQ-T	Emotion Regulation	Social Skills								
ESCS	Functional play	Social Response	Joint Attention	Receptive Language	Expressive Language	Turn-taking	Social Imitation			
EWHA-VABS	Adaptive skills	Reading	Listening	Speaking	Social Rule	Coping Skills	Social relationship			
GEM	Cognitive Empathy	Affective Empathy								
ICU	Callous-Unemotional Traits									
K-CBCL	Generic									
KBIT-II	Intelligence & Development									
KERMIT	Contextual Affect recognition									
KMAN	Affective expression recognition									
LEAS-C	Emotional Awareness									
MSEL	Intelligence & Development									
NEPSY-II: Affect recognition	Affect recognition									
NEPSY-II: Theory of Mind	Theory of Mind									
OBVL	Parenting									
POM	Pragmatic	Social initiation	Social response	Facial expression use	Facial expression response	Gesture	Body posture	Prossemic	Cooperation	Express feelings
SCAS-P	Separation anxiety	Generalized anxiety	Social phobia	Panic agoraphobia	Obsessive compulsive	Fear of physical injuries				
SPA	Symbolic Play									
SPT	Symbolic Play									
SRS-2	Social awareness	Social cognition	Social communication	Social motivation						
SRS	Social awareness	Social cognition	Social communication	Social motivation						
SSQ-P	Expression of Empathy	Sharing	Cooperation	Social Skill	Assertiveness	Self-control				
SSQ-T	Expression of Empathy	Sharing	Cooperation	Social Skill	Assertiveness	Self-control				
SSRS	Social Skill generic									
STAT	Autism generic									
TEC	Emotion comprehension	Mentalizing skills								
ToP	Social motivation	Symbolic play								
VMI	Visuo-motor integration									
VP	Visual perception									

ADOS, Autism Diagnostic Observation Schedule; BASC-2-PRS, Behavior Assessment System for Children, Second Edition- Parent Rating Scales; CAM-C, Cambridge Mindreading Face-Voice Battery for Children; CARS, Childhood Autism Rating Scale; CGI-I, Clinical Global Impression-Improvement; CSBS, Communication and Symbolic Behavior Scales; ECBI-P, Eyberg Child Behavior Inventory – Parent; EDRS, Emotion Recognition and Display Survey; ELT, Emotional Lexicon Test; ERC, Emotion Regulation Checklist; ERSSQ-P, Emotion Regulation and Social Skills Questionnaire- Parent; ERSSQ-T, Emotion Regulation and Social Skills Questionnaire – Teacher; ESCS, Early Social-Communication Scales; EWHA-VABS, Korean version of the vineland adaptive behavior scale; SCQ, Social communication questionnaire; GEM, Griffith Empathy Measure; ICU, Inventory of Callous-Unemotional Traits; K-CBCL, Korean version of the child behavior checklist; KBIT-II, Kaufman Brief Intelligence Test, Second Edition; KERMIT, Kids Emotion Recognition Multiple Images Task; KMAN, Ekman & Friesen Pictures of Facial Affect Set; LEAS-C, Level of Emotional Awareness Scale for Children; MSEL, Mullen Scales of Early Learning; NEPSY-II, A Developmental NEuroPSYchological Assessment, Second edition; OBVL, Opvoedingsbelasting vragenlijst; POM, Pragmatic Observation Measure; SCAS-P, Spence Children's Anxiety Scale—Parent; SPA, Structured Play Assessment; SPT, Symbolic Play Test; SRS-2, Social Responsiveness Scale, Second edition; SRS, Social Responsiveness Scale; SSQ-P, Social Skills Questionnaire – Parent; SSQ-T, Social Skills Questionnaire – Teacher; SSRS, Social Skills Rating System; STAT, Screening Tool for Autism in Toddlers and Young Children; ToP, Test of Playfulness; TEC, Test of Emotional Comprehension; VMI, Beery Visual Motor Integration Test; VP, Beery Visual Perceptual Subtest.

### 3.3. Study and sample characteristics

Trial size ranged from 13 to 82 participants, with four out of fourteen trials having >50 participants. The age of participants recruited ranged from 1 to 12 years. In total, 561 participants were enrolled across 14 trials. Two of this employed tablet technology with 141 participants, of which 83% were male and 17% were female. Additionally, four studies utilized computer-based interventions with 193 participants, of which 88.1% were male and 11.9% were female. Finally, eight studies utilized robotic interventions with 227 participants, of which 77.1% were male and 22.9% were female. Notably, one study did not specify the gender of participants and was therefore excluded from this demographic analysis. All studies (*n* = 14) have examined a control group (waitlist, therapy as usual or a different intervention) (Table 2).

**Table 2:** Characteristics of studies applying ICT interventions in ASC children for improving social skills.

**Tablet**						
**Reference**	**ICT system**	**Subjects**	**Social skills treatment**	**Experimental procedures**	**Outcomes measures**	**Main results**
Parsons et al. (34)	Tablet (TOBY app: Therapy Outcomes By You)	59 participants (48 M/11 F) with ASD aged between 2 and 6 years • Experimental Group (n°30) • Control Group (n°29)	• Visual motor (perception and discrimination of sensory cues) • Imitation (copying an action, design, pre-speech sounds) • Language (recognition and production of object names) • Interpersonal skills (Joint-attention)	Experimental group received therapy-as-usual and iPad + the TOBY app to practice at least 20 min daily for 3 months Control group received an iPad without the TOBY app installed and therapy-as-usual. After the waiting period of 3 months, they received the TOBY app for 3 months. Follow-up evaluation was made at 3 and 6 months for the Experimental group and at 6 and 9-months for the control group	Primary Outcomes Measures: • MSEL • CSBS Secondary Outcomes Measures: ToP POM SPT	The expressive language subscale of the MSEL was the only change between the experimental and the waitlist control group between baseline and post intervention that was statistically significant. Receptive (MSEL) and pragmatic (POM) language and social skills (CSBS) showed statistically significant improvements when the scores of all the participants were combined and tracked over time. These gains were also maintained, which suggests skill acquisition.
Kirst et al. (35)	Tablet (serious game Zirkus Empathico[ZE])	Eighty two children (69 M/13 F) aged 5–10 years with autism spectrum disorder • Experimental Group (n°42) • Control Group (n°40)	• Empathy • Emotion recognition • Emotional awareness • Emotion regulation • Callous unemotional traits • Autism social symptomatology • Wellbeing	Experimental group received manualized, tablet-based ZE intervention including 5 modules: (I) awareness of own emotions, (II) emotion recognition in faces, (III) inferring emotions from emotion-eliciting contexts (empathy), (IV) understanding emotional resonance and learning appropriate reactions toward other people's emotions, (V) fifth module emotional communication in family life. The intervention lasted for six weeks with a minimum intensity of 100 min of training per week Control group received a parent-assisted computerized training. The caregivers providing the training received different serious games, which targeted non-social skills/knowledge. The intervention lasted 100-min per week for two sessions per week The assessments took place at baseline (T1), post-treatment (T2), and at the three-month follow-up (T3).	Primary Outcomes Measures: • GEM • KERMIT Secondary Outcomes Measures: •*KMAN* • LEAS-C • ERC • ICU • SRS • Kiddy Kindl	GEM total scores for empathy and the KERMIT accuracy scores for accuracy of emotion recognition showed a between-group difference with a medium effect size favoring the experimental group at post-treatment. More long-lasting changes were observed in the experimental group for emotion regulation (ERC-ER) and the awareness of their own emotions (LEAS-C).
Beaumont et al. (36)	Computer (game-based social skills program SAS- Secret Agent Society)	Seventy child-parent dyads. Child participants (60 M/10 F) were on the autism spectrum and aged seven to 12 years • Experimental Group (n°35) • Control Group (n°35)	• Social skills • Emotional skills (i.e., emotional recognition, emotional regulation)	Experimental group received the SAS intervention consisting of Computer Games, Visual Support Cards, parent training slides, parental coaching, and Program Delivery Guide. Control group underwent a similarly structured program including a suite of engaging and interactive online cognitive activities with espionage themes but with no social or emotional skills training components. Initial parent training and weekly online coaching sessions for CIA mirrored the content and format of the SAS parent sessions The intervention lasted 30-min per week for two sessions per week over 10 weeks. The assessments took place at baseline (T0), 10 weeks after the intervention began (T1) and 6 weeks following post-intervention (T2)	Primary Outcomes Measures: • SSQ-P • ERSSQ-P • Secondary Outcomes Measures: • SSQ-T • ERSSQ-T • SCAS-P • ECBI-P	The experimental group showed significantly greater improvements in social-emotional functioning on parent-report measures (SSQ-P; ERSSQ-P) compared with those in the control group, and these were maintained at 6 weeks follow-up Parents in the experimental group reported a greater improvement in behavioral problems (ECBI-P)
Rice et al. (37)	Computer (FaceSayTM)	31 participants (28 M/3 F) with ASD aged between 5 and 11 years • Experimental Group (n°16) • Control Group (n°15)	• Emotion/Affect recognition Mentalizing/Theory Of Mind (understanding mental functions such as belief, intention, emotion, imagination • Social skills (Social Awareness, Social Cognition, Social Communication, Social Motivation, and Restricted Interests and Repetitive Behaviors) • Positive interaction (i.e., the child looks into the eyes of another child,) • Negative interaction (i.e., avoids social overtures)	Experimental Group received computer sessions utilizing FaceSayTM serious games (“Amazing Gazing”; “Band Aid Clinic,”, “Follow the Leader,”) designed to a) teach specific face-processing skills for social cognition (to attend to eye gaze and respond to joint attention); b) processing facial expressions in terms of their features (e.g., eyes and mouth) and configuration (i.e., their location on the face); and c) improve recognition and identification of emotional expressions The control group received SuccessMaker^®^, a set of computer-based courses used to improve understanding in areas such as phonological awareness, phonics, fluency, vocabulary, comprehension, and concepts of print The interventions lasted 25 min per week for a total of 10 weeks	• NEPSY-II (Affect Recognition subtest) • NEPSY-II (Theory Of Mind subtest) • SRS-2 • Behavior coding scheme and rating to assess positive/negative interactions	Children in the experimental group increase their affect recognition capabilities and mentalizing skills (NEPSY-II), and reduce their teacher-observed social impairment (SRS-2) after treatment
Hopkins et al. (38)	Computer (FaceSayTM)	49 children (44 M/5 F) with low-functioning autism (LFA) and high functioning autism (HFA) • Experimental Group (n°24) • Control Group (n°25)	• Facial recognition • Emotion recognition • Social interaction	Experimental Group. Individuals with LFA and HFA underwent social skills training with the FaceSay software twice a week for approximately 10–25 min per session over a period of 6 weeks, a total of 12 sessions Control group employed the Tux Paint, an open source drawing software for children. The interventions lasted twice a week for approximately 10–25 min per session over a period of 6 weeks, a total of 12 sessions. All post-test measures were completed within 2 weeks after the final intervention session	• KBIT, II • CARS • Six photographs of different emotional expressions contained in Unmasking the Face *KMAN* • Benton Facial Recognition Test (Short Form) • SSRS Social Skills Observation	Significant differences were found in all assessed skills between the scores obtained by the experimental group with respect to the control group. Specifically, children with LFA showed improvements in emotion recognition (Photographs) and social interactions (SSRS and • Social Skills Observation), while children with HFA demonstrated enhancements in facial recognition (Benton Short Form), emotion recognition (photos and drawings), and social interactions (SSRS and Social Skills Observation)
Thomeer et al. (39)	Computer software [i.e., Mind Reading (MR)]	Forty three children (38 M/5 F) aged 7–12 years with high-functioning autism spectrum disorder (HFASD) • Experimental Group (n°22) • Waitlist Control Group (n°21)	• Emotion recognition • Emotion decoding skills • Emotion encoding skills • Autism symptoms (Social awareness, social cognition, social communication, social motivation and autistic mannerism) • Social skills (interpersonal aspects, social adaptation and skills needed for successful interaction	Experimental group underwent MR, an interactive software program designed to teach recognition of simple and complex emotions via facial-video and vocal-audio stimuli. The program consists of 412 emotions, organized into 24 emotion groups and by 6 emotion levels. A behavioral reinforcement system was implemented to increase on task and social behaviors The children in the waitlist control group were monitored to see if they received any external clinical intervention, while the children in the treatment group were provided with the intervention Experimental group underwent 24 sessions (two 90-min sessions per week over 12 weeks and five 15–20 min intervals) Follow-up evaluations were collected after 1 week and after 5 weeks following the posttest	Primary outcome measures • CAM-C • ERDS Secondary outcome measures • SRS • BASC-2-PRS	The experimental group obtained a significantly higher score in emotional recognition in faces and voices (CAM-C) compared to the control group The results of the ERDS Receptive (decoding) and ERDS Expressive (encoding) assessments, evaluating the ability to recognize and display emotions, indicated a significant difference between the groups, favoring the experimental group ASD symptoms (SRS scores) were significantly lower after the experimental treatment with respect to traditional treatment
Marino et al. (40)	NAO robot	Fourteen children (12 M/2 F) aged 4–8 years with autism spectrum disorder • Experimental Group (n°7) • Control Group (n°7)	• Emotional recognition • Emotional comprehension and mentalizing skills • Contextual affect recognition	The experimental group underwent a robot-assisted intervention of 12 sessions (2 for the assessment and 10 for the therapy) The control group underwent the same protocol without the use of the assistive-robot, which was replaced by the therapist Each therapy session lasted 90 min and was administered twice a week, divided into four phases applied through group games and activities. First phase: emotion recognition skills Second phase: context-emotion association Third phase: discrimination between thoughts and emotions Fourth Phase: how to produce and use a repertoire of useful thoughts	• TEC • ELT	Human-assisted social robots have been found to significantly improve contextualized emotion recognition, comprehension, and emotional perspective-taking. Indeed, the experimental group showed a significantly higher degree of improvement compared to the control group for both the TEC and ELT total scores
So et al. (41)	NAO robot	13 children (10M/3F) aged 6–12 years with autism spectrum conditions • Experimental Group (n°7) • Control Group (n°6)	• Gesture use: gestural recognition and gestural production	The experimental group received robot-based gestural training into two phases. The robot taught children with ASD to recognize gestures in Phase I and produce gestures being imitated in Phase II The children in the waitlist control group watched education videos that do not contain any information about gestures in both Phases I and II The intervention program lasted for 12 weeks with each phase lasting for six weeks All the sessions in all of the phases were videotaped. Each session lasted for approximately 30 min	• VMI • VP	Individuals in the Experimental group were more likely to recognize gestures in trained and untrained scenarios. They also generalized the acquired recognition (but not production) skills to human-to-human interaction.
Yun et al., 2017 (42)	iRobiQ and CARO robots	Fifteen male aged 4–7 years with autism spectrum conditions • Experimental Group (n°8) • Control Group (n°7)	• Eye contact • Facial emotion recognition • Ability to play, general behavioral and emotional symptoms	The experimental group received robot-based intervention focused on eye contact and facial emotion recognition in three sets of interactions: (1) therapist observer– robot; (2) child–robot; and (3) therapist observer–child The control group received the training session with the human assistant that facilitated the treatment intervention. Individuals underwent eight clinical sessions, once a week, each lasting 30–40 min	Primary outcome measures • ADOS • EWHA-VABS • SCQ • SRS • Frequency of eye contact and accuracy of facial emotion expression Secondary outcome measures • K-CBCL	No significant differences between groups after treatments were detected for behavioral measures, changes in eye contact and facial emotion expression. The authors only found differences within the group after treatment
van den Berk-Smeekens et al. (43)	NAO robot	73 children (61M/12F) aged 3–8 years with autism spectrum disorder • Experimental Group 1 (n°25) • Experimental Group 2 (n°25) • Control Group (n°23)	• Social-communication skills (Social Awareness, Social Cognition, Social Communication, Social Motivation, and Restricted Interests and Repetitive Behavior) • Clinical global functioning • ASD-related symptoms	The experimental group 1 received Pivotal Response Treatment (PRT) in which parents practiced the PRT motivational techniques during parent–child interaction and were coached by the therapist The experimental group 2 received robot-assisted PRT in which a NAO robot was added in the first 15 min of each of the parent–child sessions. Motivational techniques of PRT were incorporated into game scenarios for robot-child interaction. After the robot-assisted part of the session, the session was continued as similar to the PRT condition The control group received a treatment-as-usual (TAU) consisting of guidance of parents, intensive family therapy, treatment at school (e.g., mediation), social skill training groups, pharmacotherapy, or a combination of these PRT and robot-assisted PRT consisted of 20 sessions of therapy (14 parent-child sessions, 4 parent-only sessions, and 2 teacher sessions) once a week, lasted 45 min, except for one teacher session including a 90-min school/day-care visit. The TAU ranged in intensity and frequency (from 1.5 h per week to 1 h per month)	Primary outcome measures • SRS • CGI-I Secondary outcome measures • ADOS-2 • OBVL	A significant improvement in general social-communicative skills (SRS continuous change score) at follow-up for the PRT + robot group was found with respect to the PRT group and the TAU group as rated by teachers and caregivers A higher percentage of SRS clinical responders was found for the PRT + robot group compared to other groups Finally, more children in the PRT+robot group showed a decrease in ADOS-2 severity category compared with both the PRT and the TAU groups
So et al., 2020 (44)	NAO Robots	Twenty three children (20 M/3 F) aged 4–6 years with autism spectrum disorder • Experimental Group (n°12) • Waitlist Control Group (n°11)	• Joint attention • Play behaviors	The experimental group received robot-based intervention in which any child was engaged in role-plays and practiced initiating and responding to conversations with the social robots and human experimenters The children in the waitlist control group received the same training after finishing the post-tests The treatments consisted in three training sessions for three robot dramas, with each session lasting 45 min, over 9 weeks Behavioral evaluations were completed just before the first session and 1 month after the last session of the treatment intervention	• ESCS • SPA • SRS	The children in the experimental group produced significantly more initiating joint attention behaviors in the post-test than in the pre-test (ESCS) with respect to the control group The children in the experimental group produced more other-directed functional play (SPA) after training, with respect to the control group The parents of the children in the experimental group perceived their children as having less severe social impairments after training. Indeed the children in the experimental group had significantly lower SRS scores with respect to the control group
Kim et al. (45)	PLEO robot (Social dinosaur)	24 children (21 M/3 F) aged 4–12 years with autism spectrum disorder	• Social Behavior: -Taking turns with the interaction partner - Identifying the interaction partner's emotions or expressions of preference - Shared, imaginative, and tactile play	Each participant completed a sequence of three 6-min interactional conditions, in random order: one in which the interaction partner was a dinosaur robot, another in which the partner was an adult, and a third in which the partner was a touchscreen computer game. In all three conditions, children manipulated blocks: multi-colored, magnetically linking tiles in the robot condition; multi-colored, interlocking blocks in the adult condition; and tangrams, which the participant could move and turn by dragging or tapping the touchscreen with his or her finger in the computer game condition The adult and robot interactional conditions were designed to elicit social interaction, and were semi-structured closely in parallel to each other, while the touchscreen computer game one, not The robot was pre-programmed with 10 socially expressive behaviors (made up of motor movements synchronized with speech-like vocal recordings) and three non-social behaviors. In the computer game condition, the confederate explained the goal of the tangrams game and then stopped initiating interaction, allowing the child to play the game at his or her own initiation and pace Before the first, after the final, and between conditions, each participant completed 6-min, semi-structured interview and play sessions.	Number of utterances participants produced during the interactional conditions, and to whom each utterance appeared to be directed Video recordings	Children with ASD directed a higher number of utterances to the confederate in the robot than in the adult condition, and more in both the robot and adult conditions than in the touchscreen computer game condition. There were significantly more utterances directed toward the robot and toward the adult than toward the touchscreen computer game
So et al. (46)	NAO robot	45 participants aged 4 to 6 years old, 30 of which with autism spectrum disorder • Experimental Group (n°15, two females) • Waitlist Control Group (n°15, one female) Typical development (TD; n°15, six females)	• Gestural use and production • Verbal imitation	The experimental group received robot-based gestural training for a total of 14 intransitive gestures that are commonly used in daily life. In the training sessions, the children watched the robot gesturing while narrating a set of five different stories (S1, training stories). Another set of five stories (S2, non-training stories) was presented during the assessment sessions in order to examine the generalization effects of the intervention in the novel context Both participants in the waitlist control group and participants with TD watched educational videos that were not relevant to gestural training (e.g., videos about animals) for 30 min. The waitlist control group were trained after the completion of the research The intervention program lasted for 9 weeks. It consisted of two pretests (one for each set of stories: S1 and S2), four training sessions for S1 (with two 30-min sessions per week), two immediate posttests (which were the same as the pretests), and the same follow-up posttests after 2 weeks. All the sessions were videotaped. Each session lasted for 30 min	• Video recordings • Number of gestures correctly according to four parameters: use of hand/hands, hand-shape, direction of movement, and placement. • Number of verbal imitation when they gestured	Robot-based gestural training produced intransitive gestures more accurately in training stories than those who did not receive training. Similar patterns were found in non-training stories, suggesting that the acquired gestural production skills could be generalized to novel stories. The positive learning outcomes were maintained for 2 weeks when no training was provided
Zheng et al. (47)	NAO robot	Twenty children with autism spectrum disorder • Experimental Group (n°9) • Waitlist Control Group (n°11)	• Joint attention (head pose, gaze direction)	Experimental group underwent 4 sessions of robot-mediated training to assist the child by providing prompts for joint attention. Prompts were: • pre-recorded verbal scripts • head movements to simulate gaze shifts • coordinated arm and finger pointing gestures Control group was engaged in varying levels of community treatment as usual. Each of the four experimental intervention sessions (S1–S4) lasted approximately 10 min. In each trial, a 10-s video clip was turned on contingent on the system's registration of child success or at the conclusion of the prompts. These video clips were short musical video segments of common preschool television programs	• STAT • Average prompt level that the participants needed to hit a target in a trial 2. Target hit rate, defined as the percentage of trials where participants eventually hit a target, regardless of which prompt level was needed for the target hit	Small and non-significant group differences were observed regarding improvements in response to joint attention skills within and beyond the intervention.

ADOS, Autism Diagnostic Observation Schedule; BASC-2-PRS, Behavior Assessment System for Children, Second Edition- Parent Rating Scales; CAM-C, Cambridge Mindreading Face-Voice Battery for Children; CARS, Childhood Autism Rating Scale; CGI-I, Clinical Global Impression-Improvement; CSBS, Communication and Symbolic Behavior Scales; ECBI-P, Eyberg Child Behavior Inventory – Parent; EDRS, Emotion Recognition and Display Survey; ELT, Emotional Lexicon Test; ERC, Emotion Regulation Checklist; ERSSQ-P, Emotion Regulation and Social Skills Questionnaire- Parent; ERSSQ-T, Emotion Regulation and Social Skills Questionnaire – Teacher; ESCS, Early Social-Communication Scales.

EWHA-VABS, Korean version of the vineland adaptive behavior scale; SCQ, Social communication questionnaire; GEM, Griffith Empathy Measure; ICU, Inventory of Callous-Unemotional Traits; K-CBCL, Korean version of the child behavior checklist; KBIT-II, Kaufman Brief Intelligence Test, Second Edition; KERMIT, Kids Emotion Recognition Multiple Images Task; KMAN, Ekman & Friesen Pictures of Facial Affect Set; LEAS-C, Level of Emotional Awareness Scale for Children; MSEL, Mullen Scales of Early Learning; NEPSY-II, A Developmental NEuroPSYchological Assessment, Second edition; OBVL, Opvoedingsbelasting vragenlijst; POM, Pragmatic Observation Measure; SCAS-P, Spence Children's Anxiety Scale—Parent; SPA, Structured Play Assessment; SPT, Symbolic Play Test; SRS-2, Social Responsiveness Scale, Second edition; SRS, Social Responsiveness Scale; SSQ-P, Social Skills Questionnaire – Parent; SSQ-T, Social Skills Questionnaire – Teacher; SSRS, Social Skills Rating System; STAT, Screening Tool for Autism in Toddlers and Young Children; TEC, Test of Emotional Comprehension; ToP, Test of Playfulness; VMI, Beery Visual Motor Integration Test; VP, Beery Visual Perceptual Subtest.

An extended narrative summary of the literature review is reported in the Appendix file.

### 3.3. ICT-mediated interventions: tablet

The only two studies assessing the effectiveness of behavioral interventions mediated by tablet for improving social skills abilities showed encouraging results. In the first study, Parsons et al. (34) used the TOBY app on iPad, an evidence-based and personalized intervention for ASC. The app supports four skill areas: visual-motor, imitation, language, and social (48, 49). It enhances existing therapy and can be used by families. The only statistically significant change between the experimental and control groups after 3-months of intervention was on the expressive language subscale of the MSEL. Receptive and pragmatic language and social skills showed statistically significant improvements only when all the participants' scores were combined and tracked over time, and these gains were maintained, thus suggesting skill development. In the second study, Kirst et al. (35) used a tablet game called Zirkus Empathico to help autistic children learn about emotions and empathy. The game has four modules that use videos, a mannequin, and a fox character to teach emotional awareness, recognition, cognitive empathy, and resonance. The game also suggests prosocial actions and rewards the children with circus animations. The control group played non-social games with parental help. The game improved emotion recognition and empathy more than the control group.

### 3.4. ICT-mediated interventions: computer

We found four studies assessing the effectiveness of behavioral interventions mediated by computers for improving social skills and abilities. In all papers, a significant effect of serious games was found with respect to traditional treatment. In particular, Beaumont et al. (36) used a Secret Agent Society (SAS) program to help autistic children learn social-emotional skills. The SAS has computer games, visual cards, parent training, and coaching. It teaches how to recognize emotions, use relaxation strategies, work with others, and deal with bullying. The control group played online games without social-emotional content. The SAS group improved more in social-emotional functioning and behavior than the control group. Both groups reduced anxiety.

Hopkins et al. (38) used FaceSay, a computer game with avatars, to help children with ASC learn face-processing and emotion recognition skills. FaceSay teaches how to attend to eye gaze, joint attention, facial features, and expressions. The appeal of FaceSay also lies in its ability to provide predictable results [as described by Goldsmith and LeBlanc in 2004 (50) and narrow focus (as noted by Corbett and Abdullah in 2005 (51)], made possible by interactive video-realistic avatars powered by computer technology. The control group used a drawing software. Autistic children with a co-occurring condition of intellectual disability improved more in social interactions and emotion recognition. Autistic children without intellectual disability improved more in facial recognition, emotion recognition, and social interactions.

Rice et al. (37) used FaceSay in the autistic group. The control group used SuccessMaker, a computer program for reading skills. After ten weeks, the FaceSay group improved more in affect recognition, mentalizing, and social impairment (NEPSY-II and SRS-2) than the control group.

Finally, Thomeer et al. (39) used Mind Reading (MR) software to help autistic children learn emotion recognition skills. The software has facial video and vocal-audio stimuli, and teaches 412 emotions in different levels and groups. It also has lessons, quizzes, games, and rewards. The control group had no intervention or treatment. The MR group improved more in emotion recognition of faces and voices, ASD symptom severity (SRS), and emotion display (ERDS) than the control group.

### 3.5. ICT-mediated interventions: robot

Many ICT studies that support the social skills of children with an ASD diagnosis use robot-mediated interventions. NAO is the most popular device for this purpose and was applied to improve social skills in specific domains: (a) Contextualized emotion recognition, comprehension, and emotional perspective-taking [Marino et al. (40)]; (b) Joint attention (head pose, gaze direction) [So et al. (44); Zheng et al. (47)]; (c) Gestural use/production and imitation [So et al. (41); So et al. (46)]; (d) Social Interaction [Yun et al. (42); Kim et al. (45)]; (e) Social-communication [van den Berk-Smeekens et al. (43)]; (f) Facial emotion recognition [Yun et al. (42)]; Marino et al. (40)]. Marino et al. (40) used a social robot (NAO) to help children with ASC and no intellectual disability learn socio-emotional skills. They compared 10 sessions of group cognitive behavioral therapy (CBT) with or without NAO as co-therapist. NAO taught emotion recognition, context-emotion association, thoughts-emotions discrimination, and useful thoughts. The NAO group improved more in emotion recognition, comprehension, and perspective-taking.

Regarding joint attention, So et al. (44) used NAO to help autistic children practice conversations in role-play games. The children watched and joined NAO in three dramas: “Butterfly and Farmer,” “Doctor and Patient,” and “Tourist and Tour Guide.” The NAO group improved more in joint attention and functional play than the human therapist group. The parents of the NAO group also noticed less social difficulties in their children. Zheng et al. (47) used NAO to help autistic children learn joint attention. NAO used verbal scripts, head movements, and pointing gestures to guide the children. The robot's prompts were based on the children's real-time actions. The control group had community treatment. They found no significant improvements in joint attention.

Gestural use during social communication has also been specifically investigated by So and colleagues in two different studies. In the first study [So et al. (41)], used a robot to help autistic children learn hand gestures. The robot taught them to recognize and produce eight gestures for feelings and needs. The control group had no training. The robot group improved more in gesture recognition and communication than the control group. In the second study [So et al. (46)], used a robot to help children learn intransitive gestures. The robot told stories with gestures and the children copied them. The control group had no training. The robot group made gestures more accurately than the control group. They also used gestures in new stories.

Third, social interactive behaviors generally include both verbal and nonverbal communication, such as making eye contact, using facial expressions, taking turns in conversation, and understanding social cues. In this domain, Kim et al. (45) used the social robot PLEO (dinosaur) to encourage social interaction in autistic children. The children played with blocks with PLEO, an adult, or a touchscreen game. The children talked more to the adults and PLEO than to the game. They also talked more to PLEO than to the adults. Yun et al. (42) used the iRobiQ and CARO robots to help children learn eye contact and facial emotion recognition. The children watched and joined the robots in tasks with the therapist. The control group had only the therapist. The robot group did not improve more than the control group in eye contact and facial emotion recognition.

Finally, the validity of a novel robot-mediated intervention focusing on all social communication skills (including social awareness, social cognition, social communication, social motivation, and restricted interests, and repetitive behaviors) was also a goal of van den Berk-Smeekens and colleagues' research (43). The authors used PRT, a naturalistic approach based on ABA, to help autistic children improve social skills. PRT focuses on core areas like social communication and self-initiation. The parents used PRT with their children, with or without a NAO robot. The control group had other treatments. The PRT + robot group improved more in social-communicative skills and the ADOS-2 severity rating associated with an ASD diagnosis than the other groups.

## 4. Discussion

Nearly all of the research that made up this systematic review discussed how an ICT-mediated intervention affected autistic children's social skills in meaningful ways. In fact, the vast majority of RCT studies showed a substantial effect of ICT-mediated therapies in enhancing the social abilities of autistic children, with the exception of Zheng et al. (47) and Yun et al. (42), both employed social robotic tools.

Autistic persons can learn social skills through systematic teaching, but social skills are not enough for social abilities, a broad range of socially acceptable behaviors that allow one to interact with others successfully and prevent socially inappropriate behaviors or negative reactions from other people (52). Furthermore, to achieve social competence, the child needs to interact with others in a flexible and adaptive way according to the situation (53). Therefore, autistic people need to understand the context and adapt to it, not just follow rules. This requires a wide range of specific skills that work together for flexible interaction with the environment. For this reason, a topographical representation of the targeted social abilities is essential in order to figure out the specific impact of ICT-related tools for autistic children.

**Figure 2:**
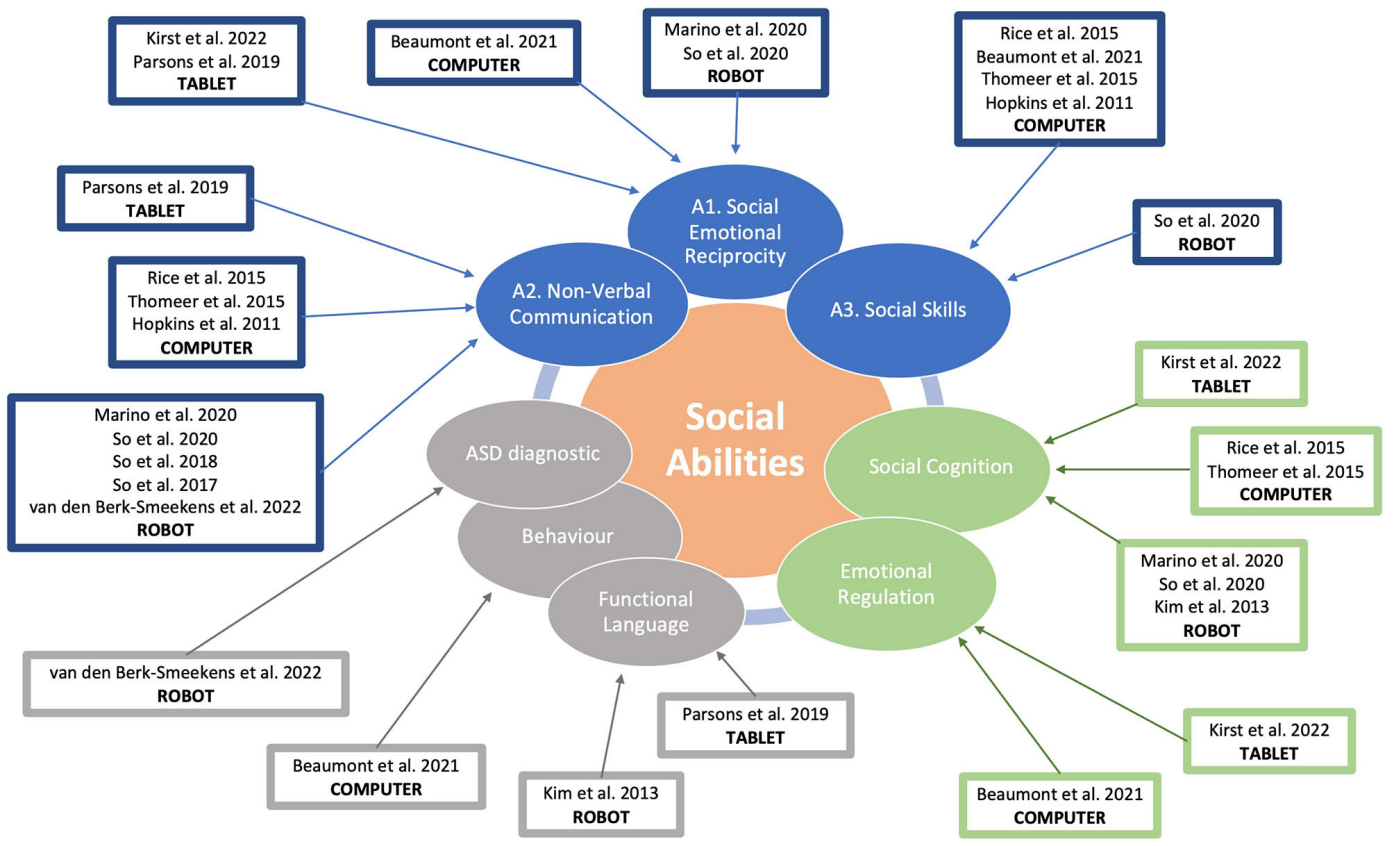
Topography of targeted social abilities by ICT tools.

In this systematic review, we categorized the 14 selected studies based on the following topics with the goal of understanding the contribution of ICT tools for ASC in terms of the specific behaviors and neurocognitive functions that they focus on enhancing. Adopting DSM-5 diagnostic criteria we can establish three different main categories: (a) social abilities as directly linked to ASD's core symptoms, (b) social abilities based on domain-specific cognitive theories, and (c) social abilities as linked to domain-general diagnoses, development, or abilities. Figure 2 shows the topography of studies for each of the main categories where ICT-related tools have been applied to improve social abilities in autistic children. The results obtained after categorizing the studies are presented in the following sections.

### 4.1. ICT applications for improving ASC social communication

Many studies targeted behaviors that are easily matched to DSM-5 areas of social communication and interaction deficits: A.1. social-emotional reciprocity; A.2. nonverbal communicative behaviors used for social interaction; and A.3. developing, maintaining, and understanding relationships. We summarized the last criteria in social skills, given that the majority of social skills programs target behaviors related to that criteria.

- A.1. The studies showed that different interventions using ICT tools improved various aspects of social-emotional skills in autistic children, such as initiating joint attention, pragmatic language, empathy, emotion awareness, social-emotional functioning, contextualized emotion comprehension and perspective-taking. The interventions used different ICT tools such as tablets, serious games or social robots (34–36, 40, 44).
- A.2. The studies showed that different interventions improved affect recognition, facial recognition, emotion recognition in faces and voices, emotion decoding and encoding. The interventions used different ICT tools such as tablets, serious games or social robots (34, 37–39).
- A.3. The studies showed that different interventions improved social understanding and functional play. The interventions used different ICT tools such as tablets, serious games or social robots (33, 36–38, 44).

We can therefore draw the conclusion that, regardless of the technologies used, ICT-mediated therapies have improved the core social communication difficulties associated with ASC in a statistically significant manner.

### 4.2. ICT applications for domain specific cognitive functions linked to social skills

Some of the studies applied a more cognitive approach or measures that allow us to point out specific constructs. Specifically the reviewed studies either looked at emotional regulation or at social cognition.

- Emotional regulation: The studies showed that the interventions improved various aspects of emotional regulation in autistic children, such as social-emotional functioning, behavioral problems, empathy, self-emotion recognition and emotion awareness. The interventions used different ICT tools such as tablets or serious games (35, 36).
- Social cognition: The studies showed that different interventions using ICT tools improved various aspects of social cognition in autistic children, such as emotion decoding and encoding, affect recognition, mentalizing skills, initiating joint attention, contextualized emotion comprehension and perspective-taking. The interventions used different ICT tools such as tablets, serious games or social robots (35, 37, 39, 40, 44).

### 4.3. ICT applications for linguistic abilities or using adaptive/maladaptive behaviors and ASD diagnostic measures

Some studies also investigated other relevant cognitive domains such as functional language, but also used generalistic behavioral scales or diagnostic instruments as outcome measures.

- Functional language: the studies showed that different interventions improved expressive language and the number of utterances. The interventions used different ICT tools such as tablets or social robots. The studies also showed that autistic children were more likely to communicate with a robot or an adult than with a touchscreen computer game (34, 45).
- Behavior: four studies used generalistic behavioral or symptom measures as the outcome. Only one (36) found a greater reduction in maladaptive behaviors and used parent reports, while the other three (39, 42, 43) found no difference.
- ASD diagnostic: It is important to highlight that three studies used ASD diagnostic measures to evaluate the outcomes of the interventions (38, 42, 43), but only one of them reported a significant change (43).

### 4.4. Commonalities and differences among different studies

The number of studies in this field is insufficient for a quantitative analysis, but we aimed to conduct a qualitative synthesis of the existing literature to identify some patterns and gaps for future research. We categorized the studies by topic-outcome and device, but other factors could also be used to compare them, such as the age of the participants, the type of framework, intervention technique, human involvement, or control group intervention.

Comparing studies on the intervention techniques or effect sizes a high level of variability can be detected. For instance, Marino and colleagues (40) developed a group cognitive behavioral therapy (CBT), based on Rational Emotive Behavior Therapy (REBT) principles, for children with typical cognitive development. In comparison to a group receiving the identical intervention without a robot, they described a broad effect size. In contrast, Yun et al. (42) focused on a very specific task (eye contact) using Discrete Trial Teaching (DTT) and they found no significant difference between the experimental and the control group. All tablet and computer interventions (34–37, 39) employed gamification procedures but in general the authors provided scarce details about the specific underlying theoretical orientation and psychological mechanism used in these protocols. For instance, in robot-mediated interventions a wide range of procedures have been described, where the vast majority used interactive play [So et al. (51); Kim et al. (47); So et al. (40); Zheng et al. (37)], but others used more specific psychological frameworks like CBT (40), PRT (43), and DTT (41, 42). Thus, in this case

However, a common trend can be observed by comparing studies by age and skill level. Null results or partial/small effects were reported mainly for studies with preschool children that trained basic skills (34, 47) or with children with intellectual disability using DTT procedures (41, 42). One possible explanation could be that children who are younger or with more cognitive difficulties, especially when trained on very specific and basic skills do not benefit from a technology mediated intervention as much as older children with typical cognitive development in the context of a more comprehensive intervention aimed at teaching higher order skills. Even when including younger children, more positive results seem to be achieved using more naturalistic interventions like PRT or CBT (40, 43). A possible explanation is that the use of Robots enhances the engagement in children who are older or have sufficient cognitive skills to perceive it as an enjoyable game and it creates a better engagement therefore facilitating learning. On the other hand, while using more structured procedures of direct teaching, especially with younger children, there is no significant difference in the interaction between a human and a robot.

### 4.5. Future directions

By categorizing the different studies (Figure 2), we can draw some useful implications for future research directions. The vast majority of reviewed studies focused only on DSM-5 criteria in the social communication and interaction domain. However, we suggest that the other criteria, such as repetitive behaviors, rigid routines, narrow interests and sensory differences, can have a significant impact on the social development and skills of autistic people. These aspects can act as either obstacles or enablers for social learning and participation. For example, repetitive behaviors may interfere with social engagement, but they may also provide comfort and predictability. Similarly, narrow interests may limit social opportunities, but they may also foster social connections with others who share them. Sensory issues may cause distress and avoidance in social situations, but they may also enhance perceptual abilities and creativity. Therefore, future studies should consider how these factors influence the social outcomes and experiences of autistic people. Future ICT intervention should focus also on those behaviors and processes to study social abilities in autism.

Another category of abilities that are relevant for autism research and intervention is cognition and functional language. These dimensions are not specific to autism, but they are known to have a significant impact on the prognosis and outcome of autistic people. They are also used in the ICD-11 classification system as criteria for subtyping autism spectrum disorder into different levels of severity and support needs.

We identified two main cognitive functions and abilities that were targeted by the studies in our categorization: emotional regulation and Theory of Mind. These abilities have been extensively researched and have played a key role in developing cognitive theories of autism and autism therapeutic interventions (54). However, there are many other cognitive theories of autism that could also inform the design and evaluation of ICT tools for autism, such as central coherence, executive dysfunction and Bayesian theories (55). For example, central coherence theories suggest that autistic people have difficulties in integrating information from different sources and levels, which could affect their social understanding and communication (56). Executive dysfunction theories propose that autistic people have impairments in planning, inhibiting, shifting, and updating information, which could affect their social flexibility and adaptation (57). Bayesian theories posit that autistic people have atypical priors or expectations about the world, which could affect their social learning and prediction (58). The use of ICT tools in interventions can allow a more direct translation from neurocognitive theory to clinical practice by providing a standardized, engaging, and adaptable way of delivering and evaluating the interventions. ICT tools, such as computers, tablets, or robots, can be designed based on the specific neurocognitive processes that are hypothesized to be impaired or atypical in autism. For example, ICT tools can be used to train executive functions by presenting tasks that require planning, inhibition, shifting, and updating information. ICT tools can also be used to measure the changes in neurocognitive processes that result from the interventions by recording behavioral or neurophysiological indicators, such as reaction time, accuracy, eye gaze, brain activity, and so on. By using ICT tools in interventions, researchers and clinicians can bridge the gap between neurocognitive theory and clinical practice and provide more evidence-based and personalized interventions for autistic people.

Some of the studies relied on diagnostic assessment, such as ADOS or CARS, or adaptive maladaptive behavior measures such as CBCL or VABS, to evaluate the outcomes of the interventions. Generalistic behavioral measures are very broad, and they may be useful at the initial stages of research to determine if there is a global improvement in positive behaviors. However, as the field of research advances, we believe that more specific measures should be favored in order to draw more refined conclusions on the specific underlying processes that are targeted by the interventions. On the other hand, using diagnostic measures as outcomes is also problematic, as they are usually too general and not sensitive enough to detect changes.

Moreover, defining a positive outcome solely based on the reduction of symptoms rather than on the enhancement of wellbeing or acquired skills is not consistent with a positive psychology perspective and can be seen as pathologizing by the autistic community. This is especially important because some autistic people can hide their social challenges (e.g., in Theory of Mind; ToM) by using their cognitive skills, and show fewer behavioral signs (e.g., on ADOS), even though they still have core difficulties. High compensators usually had higher IQ and executive functions, but also more anxiety if they also had low ToM scores. This compensation does not seem to depend on how severe the ASD diagnosis is, implying that well-compensated individuals do not have a “less serious form of ASD,” and compensation by itself may not be considered a positive outcome (59).

Another important issue for future research is how to account for the heterogeneity of the autism spectrum when comparing different tools for enhancing social skills. For example, which tool (robot, tablet) is more appropriate for autistic people with different levels of verbal ability, intellectual ability, or age? How do these factors influence the responsiveness and engagement of autistic people with these tools? Furthermore, future studies should also use consistent and reliable neuropsychological measures to assess the changes in social abilities that occur after using these tools. This would enable a more valid and comprehensive evaluation of the outcomes and benefits of these interventions for different subgroups and tailored interventions to the specific needs and strengths of different subgroups of autistic people.

### 4.6. Limitations

Despite the evidence on the effectiveness of the ICT-mediated interventions for social skills in autistic children, the translational potential of this field of study in primary care practice and educational settings is still limited by some factors, such as (a) the different duration of ICT-related interventions; (b) the high heterogeneity between psychological batteries; and (c) the reliability and validity of ICT-related tools.

One of the main aspects that could influence the outcome of ICT-mediated interventions for social skills is the duration of ICT-mediated treatments. Indeed, a wide heterogeneity characterized the literature where ICT-related training may last from 6 to 12 weeks with interventions performed once or twice a week for 30 or 60, or 100 min, without any specific reference. With respect to the neurorehabilitation of stroke or traumatic brain injury patients, in the scientific literature on ASC, there isn't strong empirical evidence of how varying treatment rigor might alter outcomes.

Another important aspect is the employment of different neuropsychological batteries to assess social skills. As reported in Table 1, to determine changes in social abilities after ICT-mediated intervention almost 39 different tests have been employed in the selected 14 RCT studies. Moreover, the vast majority of these assessments were made using parent and teacher reports rather than direct and more objective, behavioral observations. The lack of a structured, objective, shared, common neuropsychological battery to assess social skills in autistic children limited the generalizability of evidence provided in the last years about the effectiveness of treatments, reducing the possibility of comparisons between studies.

Finally, for promoting the translational potential in primary care practice of ICT interventions an objective evaluation of adherence, fidelity, and level of engagement within and among tools should be performed.

## 5. Conclusions

This review was planned and conducted following best practice guidelines for systematic reviews. From the examined studies, convergent results are found about the effectiveness of ICT-mediated interventions for improving social skills with respect to conventional face-to-face behavioral treatments. ICT is very promising for autistic people because it can reduce or eliminate several barriers that might otherwise compromise or prevent individuals with autism from actively participating in daily activities (23). Technology enables people with autism to be enabled and empowered to the same extent as their peers (60). Despite some methodological differences there is sufficient evidence to conclude that ICT-mediated interventions can be included in clinical recommendations for managing ASC-related social skill difficulties.

## Data availability statement

The raw data supporting the conclusions of this article will be made available by the authors, without undue reservation.

## Author contributions

The systematic review of literature was done by IS, FM, CF, GD, PC, RM, and NV. Drafting the manuscript was done by AC, DV, IS, AQ, and GP. Data Collection was made by LP, MC, CS, and AQ. Literature search, data interpretation, and revising the manuscript were done by IS, DV, LP, and AC. Funding resource was made by GP. All authors contributed to the article and approved the submitted version.
