# Supplementary material for: Information and communication technologies-based interventions for children with autism spectrum conditions: a systematic review of randomized control trials from a positive technology perspective
Source: Front Psychiatry. 2023 Jul 20;14:1212522. doi: 10.3389/fpsyt.2023.1212522 (PMC10398379; doi:10.3389/fpsyt.2023.1212522)
Supplement: Supplementary file 1 [file Data_Sheet_1.docx]

**Appendix: extended summary of the literature**

We report here an extended summary of the literature review. Reported references can be found in the main article.

**1. ICT-mediated interventions: Tablet**

1.1. Parsons and colleagues (34) aimed at stimulating imitation, recognition and production of object names and interpersonal skills using the iPad with the TOBY app. This app is an innovative iPad application designed to provide therapy to autistic children using evidence-based intervention strategies. The app is developed by a team of computer scientists, psychologists, and speech pathologists from Western Australia (35). The TOBY app focuses on four major skill areas that are crucial for the development of autistic children: visual-motor skills, imitation, language, and social skills (35,36). The TOBY app is not intended to replace one-on-one therapy with clinicians, but rather to complement and supplement existing therapy. It is a cost-effective and easily accessible intervention method that can be used by families to provide additional support for their child's development. The app is child-specific and personalized to the individual child's level of functioning and rate of development. It uses a curriculum that progresses based on the child's abilities, ensuring that the child is appropriately challenged and making progress towards their developmental goals. One of the major advantages of the TOBY app is that it can be used by parents and caregivers without direct input from health professionals. Instead, the autistic children enrolled in the control group received an iPad-mediated intervention without the employment of the app. The only statistically significant change between the experimental and control groups after 3-months of intervention was on the expressive language subscale of the MSEL. Receptive and pragmatic language and social skills showed statistically significant improvements only when all the participants' scores were combined and tracked over time, and these gains were maintained, thus suggesting skill development.

1.2. Kirst et al., 2022 (37) used a tablet-based intervention to stimulate several aspects of socio-emotional abilities such as, empathy, emotion recognition/awareness/regulation. The intervention was based on the serious game called Zirkus Empathico. This consists of four modules that focus on developing emotional awareness, recognition, cognitive empathy, and understanding of emotional resonance, as well as an interactive animation module for emotional communication in family life. The modules are structured into different levels, and they open up according to the child's progress within the previous module. The training is self-explanatory, with a fox character that guides the participants through the modules by providing instructions, explanations, prompts, and rewards. Training includes video stimuli that show 315 adults and children's emotional facial expressions and 62 emotion-eliciting situations filmed in the first-person perspective with an illustrated verbal introduction. Each video addresses a basic emotion, including fear, anger, sadness, surprise, joy, or a neutral state. The videos are used in different modules of the training to help the participants develop their emotional awareness, recognition, cognitive empathy, and understanding of emotional resonance. The videos provide visual and auditory cues to help the participants learn how to recognize and respond to different emotional states in themselves and others. The interactive mannequin is a key component of the game that helps children visualize and understand their own emotional states and those of others. The mannequin has two sliders that can be used to indicate bodily arousal and the valence of the child's emotions. This information is used to match the child's inner emotional state to a specific context presented using a short video clip. The child is then asked to choose an emotion label that best describes their inner state, and feedback is provided on the accuracy of their choice. The child is also asked to identify the correct emotion label for a facial expression shown by the mannequin. If the child struggles with any of these tasks, the program provides prompts and feedback to help them learn and improve. In this phase of the game, the child is encouraged to empathize with another person and to think about possible actions that can be taken to address their emotional state. The child first evaluates their own emotional state in response to the emotion-inducing context presented, using the emotions mannequin. They are then asked to identify the correct emotion label for the third person's emotional expression in the same context. If the child successfully identifies the correct emotion label, they are asked to choose between three possible courses of action: approaching the person, leaving the situation, or waiting and seeing. If the child chooses to approach the person, they are presented with a selection of concrete prosocial actions that they can take to help the person, such as being friendly, comforting, or listening. The protocol is composed of 6 or 12 levels. Each level contains 10 tasks, which are displayed as a pie chart that is filled in with the number of tasks completed. When the level is completed, the child can choose an animated reward, which is integrated into a circus arena. Extra rewards are hidden in boxes to increase motivation. In total, 30 animated reward items (e.g. bouncing ball, spinning top) and 5 animated animals can be added to the circus environment with increasing performances. Autistic children included in the control group, in contrast to the experimental group (who underwent Zirkus Empathico game), received computerized training with parental assistance. Different serious games that targeted non-social skills and information were given to the carers who were doing the training. After six weeks with a minimum intensity of 100 min of training per week, the experimental group showed better performance in the scores evaluating empathy (GEM), emotion recognition (KERMIT), emotion regulation (ERC-ER) and the awareness of their own emotions (LEAS-C) compared to the control group.

**2.ICT-mediated interventions: Computer**

2.1. Beaumont et al., 2021 (38), performed an RCT study on 35 autistic children (experimental group) undergoing a Secret Agent Society (SAS) consisting of computer games, visual support cards, parent training slides, parental coaching, and program delivery guide aimed at improving social/emotional skills. The SAS involves three different levels of activities: a) level 1 focuses on recognizing facial expressions and body posture; b) level 2 teaches players how to detect the different strengths of emotions (happy, sad, angry, and scared) within themselves through clues and body thoughts, and how to integrate facial expressions, voice, body language, and situational signals to determine how someone feels; and c) level 3 features animated missions in which the player assumes the role of an avatar and learns relaxation strategies such as breathing slowly and thinking useful thoughts. The player then takes on the role of an avatar who must work collaboratively with teammates and try out a new competitive game with them. Finally, the player assumes the role of an avatar who must initiate, maintain, and end a conversation with a group of new peers and manage to bully. Additional autistic children were enrolled as a control group and participated in a program with a similar format that included a variety of interesting and interactive online cognitive games with themes of espionage but no social or emotional skill-building exercises. In comparison to the control group, the experimental group demonstrated significantly larger gains in social-emotional functioning on parent-report measures (SSQ-P; ERSSQ-P) after 10 weeks of therapy and these were maintained at 6 weeks follow-up. Also, parents in the experimental group reported fewer behavioral issues (ECBI-P), although significant reductions in children's anxiety levels (SCAS-P) were found in both groups.

2.2. Hopkins et al., 2011 (39) tested the effectiveness of the FaceSayTM serious games on facial emotion recognition and social interaction in autistic children and with or without intellectual disability. This computer-based serious game is designed to a) teach specific face-processing skills for social cognition (to attend to eye gaze and respond to joint attention); b) process facial expressions in terms of their features (e.g., eyes and mouth) and configuration (i.e., their location on the face); and c) improve recognition and identification of emotional expressions. FaceSay uses an immersive method involving computer-generated avatars of humans and animals, to develop a software program that enhances the ability to recognize facial expressions and emotions more realistically. The appeal of FaceSay also lies in its ability to provide predictable results (as described by Goldsmith and LeBlanc in 2004 (40) and narrow focus (as noted by Corbett and Abdullah in 2005 (41)), made possible by interactive video-realistic avatars powered by computer technology. The program appears to be targeted toward promoting awareness of the movements and features of the face, particularly around the eyes. The avatar initiates interactions with the child and asks them to complete various activities involving eye gaze, facial puzzles, and facial expressions. The control group was, instead, employed in Tux Paint, an open-source drawing software for children. Children with intellectual disabilities specifically showed improvements in social interactions (SSRS and Social Skills Observation), emotion recognition (photographs), and social interactions, while children without intellectual disabilities showed improvements in facial recognition (Benton Short Form), emotion recognition (photos and drawings), and social interactions (SSRS and Social Skills Observation).

2.3. Using the same serious game, Rice et al. 2015 (42) attempted to recover theory of mind, social awareness/cognition/communicative motivation, and restricted interests in 16 autistic children, whereas the control group received SuccessMaker®, a set of computer-based courses used to improve understanding in areas such as phonological awareness, phonics, fluency, vocabulary, comprehension, and concepts of print. Following ten weeks of therapy autistic children in the experimental group improve their affect recognition and mentalizing skills (NEPSY-II), reducing their teacher-observed social impairment (SRS-2). This kind of computer-based treatment induced a robust positive effect in all assessed skills with respect to the other treatment.

2.4. Thomeer et al., 2015 (43) evaluated the impact of Mind Reading (MR) software in improving social/emotional skills. Using facial video and vocal-audio stimuli, this interactive software tool was created to teach students how to recognize both simple and complicated emotions. There are 412 emotions in the program, which are divided into 24 emotion groups and 6 emotion levels. The program has several areas that provide instruction and reinforcement, including the Emotions Library, Learning Center, Games Zone, and Rewards Zone. In the Emotions Library, children can learn about emotions through text vignettes, facial videos, and vocal-audio examples. The Learning Center offers structured lessons that use audio and visual examples to teach emotion recognition. It also includes quizzes to assess emotion recognition skills before and after completing the lessons. The Game Zone provides additional practice of emotion recognition skills through games and activities, while the Rewards Zone offers contingent access to pictures and video clips. The control group did not receive either the intervention or any external clinical treatment during the study period based on parent reports. After 24 sessions, the MR software-induced a higher score in emotional recognition of faces and voices compared to the control group, as well as a reduction of symptom severity (SRS scores). Moreover, the ability to recognize and display emotions (ERDS Receptive/Expressive (decoding/encoding) was improved in the experimental group.

**3. ICT-mediated interventions: Robot**

3.1. Marino and colleagues in 2020 (44), tested how a social robot (NAO) could help with a socio-emotional understanding intervention for autistic children and no intellectual disability. They randomly assigned 14 children aged 4 to 8 to 10 sessions of group cognitive behavioral therapy (CBT) based on Rational Emotive Behaviour Therapy (REBT) principles, either with or without a social robot’s assistance. NAO, acted as co-therapist, providing emotional and communication prompts and reinforcements with partial autonomous control. The first phase of the protocol consisted of activities focused on emotion recognition skills. The second phase addressed the teaching of context-emotion association. The third phase was centered on discrimination between thoughts and emotions. Lastly, the fourth phase aimed to provide basic strategies for and insights into how to produce and use a repertoire of useful thoughts. They used the Test of Emotional Comprehension (TEC) and the Emotional Lexicon Test (ELT) to measure the outcomes before and after the intervention. The children who received the social robot’s assistance showed significant improvements in contextualized emotion recognition, comprehension and emotional perspective-taking.

3.2. So and colleagues in 2020 (45), used NAO during role-play games aimed at stimulating children to initiate and respond to conversations with the social robots (experimental group) with respect to human therapists (control group). The children in the robot-based intervention group received nine 45-minute training sessions for three different dramas. Three drama scripts were written: "Butterfly and Farmer", "Doctor and Patient", and "Tourist and Tour Guide". The robots performed each drama twice and then invited the child to participate in a role-play. Before starting each session, one of the two robots greeted the child and gave the following instructions: "Today, we are going to perform a drama. Please sit back, relax, and watch our drama. After watching our drama twice, we will ask you to take part in a role-play with us. Do you understand?". The child played one of the characters and engaged in role-play with the robot, then switched roles and repeated the process. Compared to the control group, the children in the experimental group initiated considerably more joint attention behaviors (ESCS) and other-directed functional play (SPA), after treatment. Additionally, a significant improvement in selective performance was found in the parents of autistic children participating in the experimental group who perceived their kids had less severe social impairment (lower SRS scores).

3.3. Zheng et al., 2020 (46), instead used NAO to assist the child by providing prompts for joint attention. These prompts included pre-recorded verbal scripts, head movements to simulate gaze shifts, and coordinated arm and finger-pointing gestures. The child's actions were monitored in real-time to determine whether they were following the robot's instructions and looking at the designated monitors. The robot's prompts were then activated by an autonomous supervisory controller that controlled the interaction logic of the robotic system based on the child's real-time performance. The control group was engaged in varying levels of community treatment as usual. Investigating a small group of children, they did not report any significant improvements in joint attentional performance.

3.4 In So et al., 2017 (47), the children received robot-based hand gesture training in two phases, while the control group was assigned to a waitlist and did not receive any specific training during the study. The robot taught autistic children to recognize (Phase I) and produce specific gestures (Phase II). In particular, during Phase I the robot taught autistic children to recognize eight appropriate pantomime gestures that are frequently used in daily life to indicate feelings and needs (angry, annoyed, noisy, hot, dizzy, scared, hungry, smelly). Children's abilities to recognize gestures in different scenarios were evaluated in four pre-tests and the robot asked the child to choose the correct gesture that represented the scenario. During Phase II children were asked to produce appropriate gestures in different scenarios. Four pre-tests were conducted, and the child was asked to demonstrate a gesture that matched a scenario. After 12 weeks of treatment, in both trained and untrained sessions, people in the experimental group were more likely to recognize gestures and apply the learned recognizing (but not production) skills to interpersonal communication.

3.5. So et al., 2018 (48) enrolled 30 autistic children (randomly assigned to the Experimental/Control group) with respect to 15 children with typical development (TD). The experimental group underwent robot-based gestural training for a total of 14 intransitive gestures that are commonly used in daily life (e.g., both hands clapping means awesome; two arms open wide with palms facing up means where; a hand waving means bye; opening arms wide means to welcome to others.). During this training, children observed the robot narrating a collection of five distinct stories while making gestures (training stories). During the evaluation sessions, a different set of five tales (non-training stories) were given to test the generalization effects of the intervention in the novel setting. After 9 weeks of treatment, autistic children who had robot-based gestural instruction generated intransitive gestures more accurately than autistic children who did not. Similar trends were observed in stories that were not part of training, indicating that the gestural production skills could be applied to new stories.

3.6. Kim et al., 2013 (50) used the social robot PLEO (dinosaur), designed to elicit social interaction, that was pre-programmed with 10 socially expressive behaviors and three non-social behaviors. A group of 24 autistic children was observed during three interaction conditions (presented in random order) led and facilitated by an adult confederate. The interaction partner in each of the three situations varied: either a touchscreen computer game, a second adult person, or the social robot PLEO. In all three conditions, children were engaged in block manipulations: a) in the robot scenario, they used multicolored, magnetically linking tiles; b) in the human-mediated scenario, they used multicolored, interlocking blocks; and c) in the computer game scenario, they used tangrams that they could move and turn by dragging or tapping on the touchscreen. A significant increase in the total number of utterances directed to the confederate was detected in the robot condition than in the adult one, and more in both the robot and adult conditions than in the touchscreen computer game condition. Furthermore, there were significantly more utterances directed toward the robot and toward the adult than toward the touchscreen computer game.

3.7. Yun et al., 2017 (49) used the iRobiQ and CARO robots to stimulate the recovery of eye contact and the ability to play and recognize facial emotion. Using a small sample of children, they engaged autistic children in the experimental group in three different sets of interactions (centered on eye contact and facial emotion recognition): a) therapist observer-robot, b) child-robot, and c) therapist observer-child. Whereas the control group only received the training session with the human assistant that facilitated the treatment intervention. The eight-session intervention program was based on the discrete trial teaching protocol and focused on eye contact and facial emotion recognition. Ten tasks were incorporated into each session to practice gaze and facial emotion recognition. The first part of each session consisted of a training phase, in which the child attended a short interaction in which the therapist correctly responded to the robot's request to identify his emotional expression. This served to facilitate the child's involvement and imitation in future interactions. During the next practical phase, the robot addressed the child, greeting him and trying to establish eye contact with him before proceeding with the facial emotion recognition tasks. Then the robots presented a series of different emotional facial expressions and asked the child to identify them while the therapist recorded the answers as right or wrong. During the robotic interactions, four modules occurred: training element query, recognition of human activity, coping-mode selection, and follow-up action. After treatment, no significant changes in Goldstein's eye contact and facial emotional expression were found in the experimental group with respect to the control group.

3.8. van den Berk-Smeekens and colleagues' research (51) tested the validity of a novel robot-mediated intervention focusing on all social communication skills (including social awareness, social cognition, social communication, social motivation, and restricted interests, and repetitive behaviors). In this work, the behavioral intervention was mediated (or not) by social robots using the Pivotal Response Treatment (PRT) approach. The PRT is a behavioral intervention that focuses on "pivotal" (core) areas to improve other areas of functioning and skills. These pivotal areas include social communication, responsiveness, self-initiation, and self-management. PRT is a naturalistic approach based on ABA. In the parent-only condition, parents interacted with their children while using PRT motivational tactics, while the therapist provided coaching. Instead, the second experimental group received robot-assisted PRT, which included the addition of an NAO robot for the first 15 minutes of each parent-child session. Gameplay scenarios for robot-child interaction included PRT's motivational strategies. The session was continued in a manner similar to the PRT condition after the robot-assisted portion. Finally, the control group underwent the treatment-as-usual (TAU) condition, in which social skill training groups, medication, intense family therapy, parental supervision, treatment at school (such as mediation), and/or a combination of these were included. After 20 sessions of therapy, a significant improvement in general social-communicative skills (SRS continuous change score) and a decrease in the ADOS-2 severity category was found for the PRT + robot group with respect to other groups.
